# CDK6 Immunophenotype Implicates Potential Therapeutic Application of CDK4/6 Inhibitors in Urothelial Carcinoma

**DOI:** 10.3389/fonc.2022.819003

**Published:** 2022-04-08

**Authors:** Ran Sun, Xuemei Wang, Leichao Zhang, Yu Gu, Shaojuan Yang, Liping Wang, Xueju Wang

**Affiliations:** ^1^Center for Reproductive Medicine, China-Japan Union Hospital, Jilin University, Changchun, China; ^2^Department of Pathology, China-Japan Union Hospital, Jilin University, Changchun, China

**Keywords:** CDK6, urothelial carcinoma, CDK4/6 inhibitor, precision medicine, immunohistochemistry staining

## Abstract

**Background:**

Infiltrating bladder urothelial carcinoma is the most common bladder malignancy with limited therapeutic options and poor prognosis. Identifying new therapeutic targets or strategies has important clinical significance. The data from public sources indicate poor prognosis in urothelial carcinoma cases with high CDK6 mRNA levels. Furthermore, studies have shown that CDK6 expression is elevated in urothelial carcinoma tissue compared to the surrounding urothelium, thus presenting a case for performing CDK4/6 inhibitor targeted research in urothelial carcinoma. However, a phase II trial showed that CDK4/6 inhibitors are not effective for advanced urothelial carcinoma, suggesting that case screening is important for targeted therapy.

**Objective:**

Immunohistochemistry (IHC) is simple and easy to perform and can be used to screen urothelial carcinoma cases with high CDK6 expression in clinical practice. The aim of this study was to determine the CDK6 expression threshold for positive cases.

**Methods:**

We evaluated the correlation between the H-score of CDK6 protein expression and survival or CDK6 mRNA level using RNA sequencing. The effects of different CDK4/6 inhibitors were tested on bladder carcinoma cell lines with different CDK6 expression levels.

**Results:**

The H-score, which predicts poor prognosis and reflects a high CDK6 mRNA level, was determined as the selection criterion for positive cases. Furthermore, we found that urothelial carcinoma cell lines with higher CDK6 expression levels displayed greater sensitivity to CDK4/6 inhibitors than cells with lower expression levels.

**Conclusions:**

IHC staining for CDK6 protein in urothelial carcinoma is proposed as a promising screening platform for CDK4/6 inhibitor targeted therapy.

## Introduction

Infiltrating urothelial carcinoma is the most common bladder carcinoma (BLCA), with limited therapeutic options and poor prognosis (1). Conventional BLCA is typically divided into low- and high-grade lesions, depending on the architecture disorder, nuclear pleomorphism, and pathological mitoses. High-grade invasive BLCA presents remarkable diversity in morphological features, such as a wide range of architectural patterns, variegated cytological changes, and more aggressive behavior than low-grade counterparts. Approximately one-fourth of high-grade BLCA cases are accompanied by histological variants, such as squamous, nested, or plasmacytoid differentiation (2). Although several histological variants of BLCA are listed in the 2016 World Health Organization classification of urothelial tract tumors (3, 4), there remains controversial evidence on the influence of these histological variants on the prognosis and response to BLCA treatment because of the limited number of cases involved (2, 4). Furthermore, an increasing number of molecular subtypes have been defined in BLCA; basal and luminal subtypes are the most stably accepted subtypes and display the upregulation of KRT5, KRT6, or GATA3 genes, and it is well known that BLCA with basal subtypes predicts worse prognosis (5).

Cell cycle progression is promoted by CDK6 through interaction with D-type cyclins to phosphorylate the retinoblastoma tumor suppressor and displays oncogenic potency by kinase activity in several types of malignant tumors (6–8). A study involving 31 urothelial carcinoma cases showed higher CDK6 expression levels in the BLCA tissues than in adjacent non-neoplastic tissues (9), suggesting that CDK6 could be a potential therapeutic target for urothelial carcinoma. CDK4/6 inhibitors have been approved by the US Food and Drug Administration for the treatment of postmenopausal women with advanced breast cancer (10) and have been involved in the clinical trial of several solid tumors and lymphomas (11). Research has shown that CDK4/6 inhibitors are active as a novel therapeutic approach *in vitro* and *in vivo* in bladder cancer cells (12). However, a phase II clinical trial showed that the CDK4/6 inhibitor palbociclib does not demonstrate meaningful activity in 12 selected patients with platinum-refractory metastatic urothelial carcinoma (13). In this study, cases with p16 loss and Rb intact immunophenotype were included, CDK6 expression levels were ignored (13). Actually, independent of kinase activity, CDK6 also demonstrate transcriptional regulation activity in malignant tumor (14). Thus, it is imperative to explore the relationship between CDK6 expression and therapeutic options in BLCA.

We investigated the Cancer Genome Atlas (TCGA) and GEO databases and found that high CDK6 transcriptional levels were correlated with poor prognosis, and more frequently accumulated in muscle-invasive bladder cancer (MIBC) than in non-muscle-invasive bladder cancer (NMIBC) cases. Thus, it is worthwhile to evaluate CDK6 gene expression in BLCA cases for the potential application of CDK4/6 inhibitor target therapy. The evaluation of mRNA levels is relatively more expensive and time-consuming than immunohistochemistry (IHC) staining in clinical practice (15, 16). Both increased cytoplasmic and nuclear distributions of CDK6 protein are observed in several solid tumors by IHC method. In these studies, nuclear distribution of CDK6 protein is more objectively evaluated than cytoplasmic distribution to associate with unfavorable prognosis (7, 9, 17). The goal of this study was to establish a screening platform by IHC staining of CDK6 protein to predict prognosis and potential opportunities for CDK4/6 inhibitor treatment in BLCA.

## Materials and Methods

### Study Design

Eighty-five patients diagnosed as primary BLCA by transurethral resection or radical cystectomy or cystoprostatectomy from June 2010 to June 2014 at the China-Japan Union Hospital of Jilin University (Changchun City, Jilin Province, People’s Republic of China), were retrospectively selected for the study. Related clinicopathological information was obtained by an investigator blinded to the other results. Histological grading was briefly classified as low-grade or high-grade. *In situ* cancer should display a high-grade morphology. Cancer staging was performed according to the American Joint Committee on Cancer 8th Edition manual. Detailed clinicopathological descriptions of all cases are presented in **Table 1**. Formalin-fixed and paraffin-embedded (FFPE) specimens of 85 patients were used for IHC studies, of which 22 fresh frozen tissue samples stored immediately at −80°C in a tissue bank were used for RNA sequencing experiments. This study was approved by the Institutional Medical Ethics Review Board of the China-Japan Union Hospital of Jilin University in compliance with the Declaration of Helsinki. The reference number was 2021-KYLL-030003. All patients gave informed written consent for the provision of a tumor sample.

**Table 1:** Clinical-pathological information of 85 bladder carcinoma (BLCA) patients in this study.

Case No.	Age	Gender	Surgery	AJCC staging*
1	70	M	RC	II
2	48	M	RC	III
3	71	M	TUR	II
4	69	M	RC	IV
5	47	M	RC	IV
6	56	M	RC	IV
7	49	F	TUR	0is
8	73	M	TUR	I
9	69	M	TUR	I
10	65	F	TUR	I
11	69	M	RC	III
12	64	F	TUR	IV
13	37	M	TUR	0a
14	42	M	TUR	I
15	73	M	TUR	I
16	75	F	TUR	I
17	66	M	RC	II
18	56	F	TUR	0is
19	62	M	RC	III
20	65	M	TUR	I
21	77	M	TUR	IV
22	86	F	TUR	II
23	60	M	TUR	I
24	78	M	TUR	IV
25	55	M	TUR	0is
26	86	M	TUR	I
27	58	M	TUR	0a
28	63	M	TUR	0
29	76	M	TUR	0
30	54	M	TUR	I
31	60	M	TUR	0
32	53	M	RC	II
33	66	M	TUR	I
34	64	M	TUR	IV
35	70	M	RC	II
36	57	M	TUR	II
37	87	M	TUR	0
38	54	M	RC	I
39	61	M	RC	III
40	54	F	TUR	0
41	74	M	RC	III
42	69	M	RC	I
43	53	M	TUR	0a
44	73	M	RC	I
45	53	M	RC	III
46	53	M	TUR	I
47	43	M	TUR	I
48	60	F	TUR	0is
49	49	M	TUR	0a
50	41	M	TUR	II
51	72	M	TUR	0a
52	68	M	TUR	0a
53	77	F	TUR	0is
54	52	M	RC	III
55	70	F	TUR	I
56	70	M	RC	II
57	56	F	TUR	0a
58	67	M	RC	III
59	65	M	TUR	II
60	84	M	RC	IV
61	63	M	TUR	I
62	59	M	TUR	I
63	71	F	TUR	0is
64	38	M	TUR	IV
65	64	M	RC	I
66	60	M	TUR	0is
67	63	M	TUR	I
68	68	M	RC	IV
69	73	M	TUR	I
70	61	M	TUR	0a
71	74	F	TUR	IV
72	57	M	TUR	I
73	85	M	TUR	I
74	72	M	TUR	I
75	48	M	RC	IV
76	67	M	RC	III
77	65	M	RC	III
78	51	M	RC	II
79	73	M	RC	IV
80	46	M	RC	IV
81	67	M	RC	II
82	55	M	RC	III
83	76	F	RC	IV
84	64	M	RC	I
85	64	M	RC	I

RC, radical cystectomy or cystoprostatectomy; TUR, transurethral resection; *American Joint Committee on Cancer (AJCC) 8th Edition.

### Analysis of GEO and TCGA Databases

The gene expression profile of GSE13507 was downloaded from the GEO database (https://www.ncbi.nlm.nih.gov/). GSE13507 included 165 primary bladder cancer samples, of which 62 were MIBC cases and 103 NMIBC cases. Another expression profile of BLCA samples from the TCGA database was analyzed using UALCAN-UAB (http://ualcan.path.uab.edu) (18). TCGA data consisted of 406 cases, with most cases in the advanced stage (n = 403). Both GSE13507 and TCGA data had complete corresponding clinical information. Comparison of CDK6 expression between MIBC and NMIBC was evaluated using GSE13507. Comparison of CDK6 expression among advanced stages and cancer-specific survival analysis was performed in both GSE13507 and TCGA.

### IHC Staining

The IHC assay was performed as previously described (19). The monoclonal mouse anti-CDK6 antibody (1:200 dilution; ab124821, Abcam, Cambridge, MA, USA) was used for primary antibody incubation at 4°C overnight. A slide incubated without the primary antibody was used as a negative control. A secondary antibody was applied using the Elivision Plus Kit (Dako, Glostrup, Denmark) according to the manufacturer’s instructions.

### IHC Evaluation

All staining slides were blindly and independently reviewed by three pathologists for scoring CDK6 nuclear staining. The staining intensity was scored as 0, 1 (weakly positive), 2 (moderately positive), and 3 (strongly positive). The percentage of positive cells was scored as 0, 1 (< 5% positive), 2 (5%–50%), and 3 (> 50%). The H-score was calculated as the product of the multiplication of the percentage of area stained at each intensity level multiplied by the weighted intensity (20). The H-scores ranged from 0 to 300. The median H-score was 210.

### RNA Sequencing

RNA sequencing was performed on total RNA extracted from fresh frozen tissue samples of 22 BLCA cases. The RNA Nano 6000 Assay Kit of the Bioanalyzer 2100 system (Agilent Technologies, Santa Clara, CA, USA) was used to assess RNA integrity. A total of 1 μg RNA per sample was used as the input material for the RNA sample preparations. Clustering of the index-coded samples was performed on a cBot Cluster Generation System using TruSeq PE Cluster Kit v3-cBot-HS (Illumina) according to the manufacturer’s instructions. After cluster generation, the library preparations were sequenced on an Illumina Novaseq platform and 150 bp paired-end reads were generated. Normalized read count data and fragments per kilobase of exon per million reads (FPKM) were used to evaluate gene expression.

### Cell Lines and Cell Culture

The T24 and 5637 cells (human bladder cancer cell lines) were obtained from the American Type Culture Collection and grown in RPMI 1640-medium supplemented with 10% fetal bovine serum (Hyclone), 2 mM glutamine, and antibiotics (100 U/mL penicillin and 100 μg/mL streptomycin) at 37°C in a humidified 5% CO2 atmosphere.

### CDK4/6 Inhibitors and Proliferation Inhibition Assay

To investigate the potential inhibitory effects of CDK4/6 inhibitor on T24 and 5637 cells, we tested the inhibitory effect of two CDK4/6 inhibitors, ribociclib (Cat No: HY15777, MCE) and palbociclib (Cat No: HY50767, MCE) on human bladder carcinoma cells. The T24 or 5637 cells (1.5 × 10^4^ cells per well) were plated in a 96-well plate, and the cells were treated with ribociclib or palbociclib alone at concentrations from 0 to 50 μM for 72 h at 37°C. Cell proliferation was determined by an assay using the Cell Counting Kit-8 (DojinDo, cat#:CK04) and expressed as mean A450 value (absorbance value) ± standard deviation (SD) of triplicate wells. GraphPad Prism software version 8.3.0 (GraphPad, Inc., San Diego, CA, USA) was used to calculate IC50 values and create a relative inhibition curve. The experiments were independently repeated three times. The mean IC50 value ± standard error of the mean (SEM) of three independent experiments was used for statistical analysis.

### Western Blotting

Cells were collected using radioimmunoprecipitation assay buffer (Thermo Scientific, cat# 89901) supplemented with protease inhibitors, phosphatase inhibitors, and PMSF, all of which were purchased from Boster Biotech, China. Protein concentrations were determined using the Pierce BCA Protein Assay Kit (Thermo Scientific, cat# RD231228). The total proteins were separated on a 10% sodium dodecyl sulphate-polyacrylamide gel and then transferred to a nitrocellulose membrane (AR0135-02, Boster, China). After blocking the membrane in 5% (w/v) Difco Skimmed Milk (Biotopped, China) for 2 h at 15–25°C, the membranes were incubated with antibodies against CDK6 (ab124821, Abcam, Cambridge, MA, USA) and tubulin (#2148, CST, USA) at a concentration of 1:5000 at 4°C overnight. The membranes were washed three times with PBS/T and visualized using the LI-COR Odyssey Detection Kit.

### Follow-Up

The follow-up period of the patients was at least 6 years after the initial pathological diagnosis of BLCA. Recurrence and BLCA-specific survival were recorded for statistical analyses in this study. Patients who died owing to causes other than BLCA were excluded.

### Statistical Analysis

A non-parametric test was performed to compare the distribution of CDK6 expression between the NMIBC and MIBC groups. The correlation between CDK6 expression and clinical-pathological features was determined by the Fisher’s exact test. The R project (using “survminer” packages) was used to determine the optimal cutoff value of CDK6 levels for prognostic and cancer-specific survival analysis (21, 22). Log-rank (Mantel–Cox) and Gehan–Breslow–Wilcoxon tests were applied to verify the survival analysis results. Correlation analysis between the H-score and gene expression designated either by gene counts or FPKM was performed using Pearson correlation analysis. Statistical analysis of cell proliferation was performed using the Student’s *t*-test. Values of *p* < 0.05 were considered significant.

## Results

### High CDK6 Transcriptional Level Is Associated With Advanced Stage and Poor Prognosis in BLCA Cases Obtained From a Public Database

**Figure 1:**
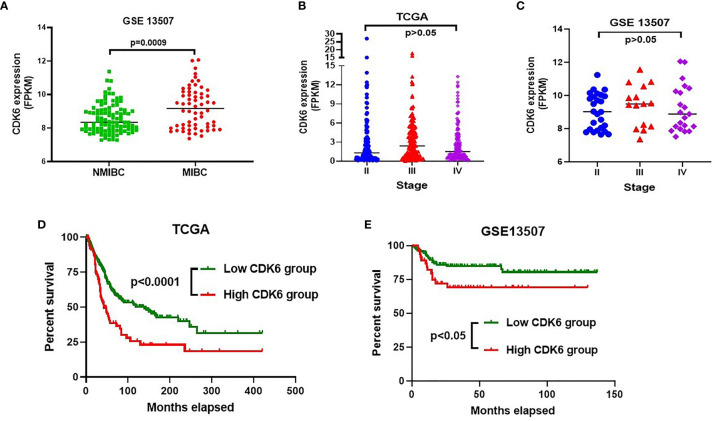
CDK6 transcriptional level analysis in bladder carcinoma (BLCA) from GEO and TCGA database. Muscle-invasive bladder cancer (MIBC) group displayed significantly higher CDK6 level than that in non-muscle-invasive bladder cancer (NMIBC) group (*p*<0.001) **(A)**. There was no difference on the CDK6 transcriptional level through stage II to IV by analyzing both GSE13507 **(B)** and TCGA **(C)** cohorts (*p*>0.05). High CDK6 transcriptional cases in 93 (23%) out of 406 cases from TCGA database displayed worse prognosis than that in low level group (n=313) (*p*<0.001) **(D)**. Similarly, CDK6 transcriptional cases in 48 (29%) out of 165 cases also showed worse prognosis than that in low level group (n=117) in GSE13507 cohort (*p*<0.05) **(E)**.

To observe the relationship between CDK6 expression and cancer progression, we compared the transcriptional level of CDK6 between MIBC (n = 62) and NMIBC (n = 103) in the GSE13507 cohort (including 165 BLCA cases). As shown in **Figure 1A**, there were significantly higher CDK6 levels in MIBC than in NMIBC (*p* < 0.001). There were also no differences in the CDK6 transcriptional level of the TCGA and GSE13507 cohorts from stages II to IV (**Figures 1B, C**). Furthermore, the high-CDK6 transcriptional cases in 93 (23%) out of 406 cases from the TCGA database displayed worse prognosis than those in the low-level group (n = 313) (*p* < 0.001, **Figure 1D**). Similarly, CDK6 transcriptional cases in 48 (29%) out of 165 cases also showed worse prognosis than those in the low-level group (n = 117) in the GSE13507 cohort (*p* < 0.05, **Figure 1E**). Based on these results, we hypothesized that CDK6 was associated with cancer progression from the NMIBC to MIBC stage and approximately one-fourth of BLCA cases (23% in TCGA database and 29% in GSE13507 cohort), displayed high CDK6 transcriptional levels correlated with reduced survival. Thus, it is worthwhile to detect transcriptional CDK6 levels to predict prognosis and evaluate target therapy potential in BLCA cases. However, mRNA detection is relatively expensive and complicated for clinical practice; thus, it is important to develop a simplified screening platform.

### Correlation of CDK6 Expression and Clinical-Pathological Features in BLCA Cases

**Figure 2:**
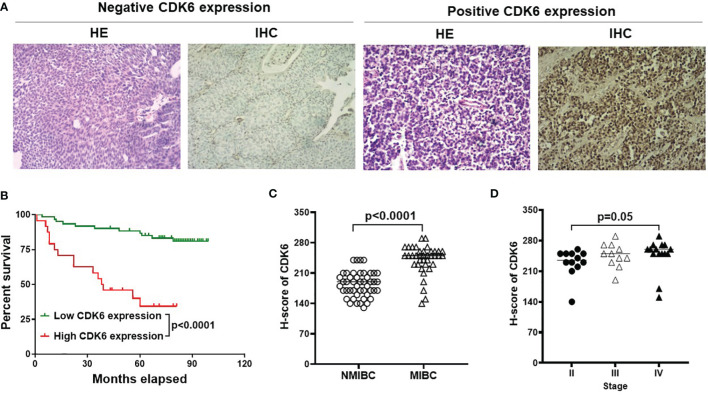
IHC for CDK6 expression in BLCA. Representative images of H&E and IHC staining from negative and diffusely strong positive cases [**(A)**, Magnification, X40]. Cases with high H-score was correlated with worse prognosis compared with low or no expression group [*p* < 0.0001, **(B)**]. Furthermore, MIBC group (n=38) displayed higher CDK6 expression compared to NMIBC group (n=47) [*p* < 0.0001, **(C)**], but there was no difference among advanced stages [from stage II to IV, *p* = 0.05, **(D)**].

To simplify the validation of CDK6 expression in routine clinical practice, FFPE tissues from 85 BLCA cases in the pathology department of the China-Japan Union Hospital were used in this study for IHC staining of CDK6 protein. Representative images of negative and diffusely strong positive staining for CDK6 are shown in **Figure 2A**. Although there were no significant differences in the distribution of age, sex, histological grade, and recurrence rate between groups with either high or low CDK6 H-scores (p > 0.05, **Table 2**). The best cutoff value of H-score was calculated by R project (21, 22). The BLCA cases with H-scores higher than 240 (n = 24) accounted for 28% (24/85), which displayed increased metastasis potential (*p* < 0.0001, **Table 2**) and worse prognosis (*p* < 0.0001, by both the log-rank (Mantel–Cox) test and the Gehan–Breslow–Wilcoxon test; **Figure 2B**) than those with H-scores less than or equal to 240 (n = 61). Cases with high CDK6 H-scores were accumulated more in MIBC group (n = 38, including stage II to IV) than the NMIBC group (n = 47, including stage 0 to I) (p < 0.0001, **Figure 2C**). Furthermore, there was no difference in MIBC group among the advanced stages (n=38, from stage II to IV, p > 0.05, **Figure 2D**). These data suggest that CDK6 is involved in the advance stages (from stage II to IV) of BLCA and meaningful to be detected by IHC for evaluating prognosis and therapeutical potential of CDK4/6 inhibitors in these populations.

**Table 2:** Correlation of CDK6 expression and clinical-pathological features in Bladder carcinoma (BLCA).

BLCA Patients	Numbers	CDK6 expression		*p* values
		Low	High	
Age (years)				
Median	64	64	64	>0.05
Range	37-87	37-87	41-86	
Sex				
Male	71	51	20	>0.05
Female	14	10	4	
Histological grading				
Low grade	8	8	0	>0.05
High grade	77	53	24	
Recurrence				
No	40	32	8	>0.05
Yes	45	29	16	
Metastasis				
No	68	58	10	<0.0001^*^
Yes	17	3	14	

*Means that Values of p were considered significant.

### Correlation Between Protein and mRNA Levels of CDK6 Expression in BLCA Cases

**Figure 3:**
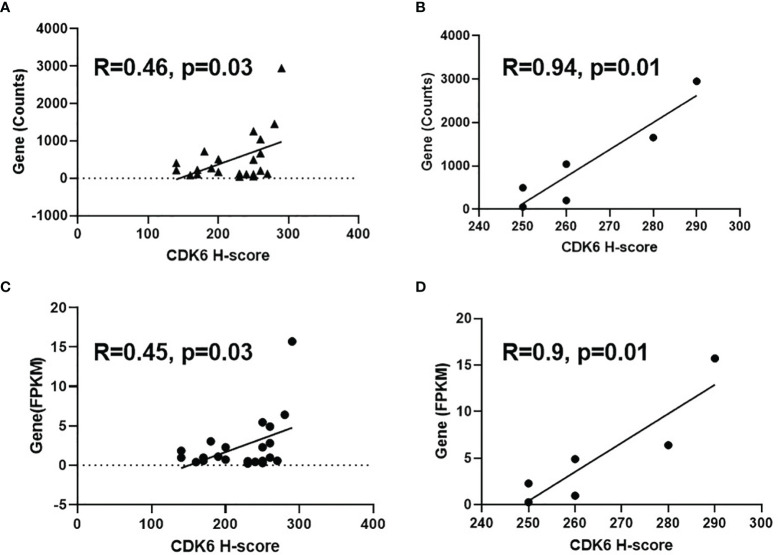
Correlation between IHC score and mRNA level of CDK6 expression. Moderate correlation was observed [R=0.46, *p*=0.03 by H-score vs gene counts, **(A)**; R=0.45, *p*=0.03 by H-score vs FPKM value, **(C)**] for the comparison of H-score and mRNA level of 22 cases. Strong correlation was shown between IHC and RNA-sequencing methods [R=0.94, *p*=0.01 by IHC scores vs gene counts, **(B)**; R=0.9, *p*=0.01 by IHC scores vs FPKM value, **(D)**] after narrowing to the cases with CDK6 scores higher than 240.

To observe the consistency between protein and mRNA levels of CDK6 in BLCA cases, we further conducted RNA sequencing on 22 cases and compared either gene counts or FPKM values to the CDK6 H-score. As shown in **Figures 3A, C**, a moderate correlation was observed (R = 0.46, *p* = 0.03 by H-score vs. gene counts; R = 0.45, *p* = 0.03 by H-score vs. FPKM value). Narrowing to cases with CDK6 scores higher than 240, there was a strong correlation between IHC and RNA-sequencing methods (R = 0.94, *p* = 0.01 by IHC scores vs. gene counts; R = 0.9, *p* = 0.01 by IHC scores vs. FPKM value; **Figures 3B, D**), suggesting that high CDK6 H-score level is consistent with high CDK6 mRNA levels in BLCA cases.

### CDK6 Protein Level Was Correlated to CDK4/6 Inhibitor Effect in BLCA Cell Lines

**Figure 4:**
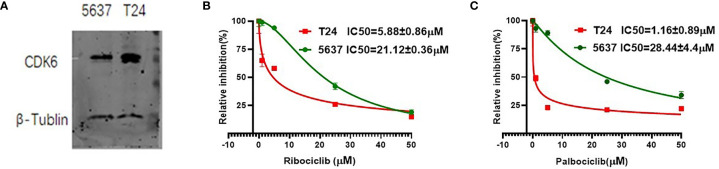
The CDK6 protein expression and IC50 value of CDK4/6 inhibitors in urothelial carcinoma cell lines. Western blot analysis showed higher CDK6 protein expression in T24 cell lines compared to 5637 cell lines **(A)**. Relative growth inhibition curves of ribociclib **(B)** and palbociclib **(C)** in 5637 or T24 cell lines were from three independent experiments. Mean IC50 value of ribociclib was 21.12 ± 0.36 μM for the 5637 cells and 5.88 ± 0.86 μM for the T24 cells (p <0.001), and mean IC50 value of palbociclib was 28.44 ± 4.4 μM for the 5637 cells and 1.16± 0.89 μM for the T24 cells (p = 0.001).

We selected two urothelial carcinoma cell lines, T24 and 5637, with different CDK6 protein expression levels (**Figure 4A**), to observe the inhibitory effect of CDK4/6 inhibitors. The T24 or 5637 cells were incubated for 72 h with the CDK4/6 inhibitors ribociclib or palbociclib at a series of concentrations and the IC50 value of each drug on each cell line was calculated. Both ribociclib and palbociclib significantly inhibited the proliferation of 5637 and T24 cells in a dose-dependent manner. As shown in **Figures 4B, C**, mean IC50 value of ribociclib was 21.12 ± 0.36 μM for the 5637 cells and 5.88 ± 0.86 μM for the T24 cells (*p <*0.001), and mean IC50 value of palbociclib was 28.44 ± 4.4 μM for the 5637 cells and 1.16± 0.89 μM for the T24 cells (*p* = 0.001). The IC50 of ribociclib in the 5637 cells was almost four times higher than that in the T24 cells, and the IC50 of palbociclib in the 5637 cells was approximately 28 times higher than that in the T24 cells. The T24 cells displayed higher CDK6 protein expression levels and more sensitivity to CDK4/6 inhibitors than those of the 5637 cells, suggesting that sensitivity to CDK4/6 inhibitors is accompanied by CDK6 protein expression levels in BLCA.

## Discussion

The IHC of CDK6 protein may be used for predicting prognosis in BLCA cases, especially those with high-grade morphology. In this study, we found that both high mRNA level and high H-score of CDK6 protein correlated with poor prognosis in BLCA. However, only 85 cases were evaluated for CDK6 protein IHC staining. We should increase the number of cases, especially cases in advanced stages to further validate the prediction effect of IHC staining in the future. Furthermore, the basal phenotype displayed worse prognosis than the luminal phenotype in BLCA and Guo et al. developed a routine IHC staining of GATA3 and KRT5/6 to designate basal and luminal molecular subtypes, respectively, for routine clinical practice (5). This provides us with a further research direction for identifying the relationship between the CDK6 protein and molecular phenotype. As a next step, we may observe the relationship between the expression of CDK6 and GATA3 or KRT5/6 by IHC staining to determine whether CDK6 gene expression is the signature of the basal phenotype.

CDK4 and CDK6 are known as homologous enzyme in cell cycle regulation. In this study, we observed the activity of CDK6 in urothelial carcinoma cases and defined about 28% (24/85) CDK6 high expression cases with unknown CDK4 state. It will be meaningful to explore CDK4 level in cases with either CDK6 high level or low level and evaluate the influence of CDK4 expression on the sensitivity to CDK4/6 inhibitors in BLCA in the next step. Furthermore, since CDK6 display both kinase activity in cell cycle regulation and transcriptional regulation activity (14), it will be useful to investigate the oncogenic mechanism of CDK6 in urothelial carcinoma by either knockdown or overexpression of CDK6 *in vitro* experiment in the future.

As CDK6 participates in cell cycle dysregulation in many solid tumors and lymphoma (6, 8), there is a potential benefit of CDK4/6 inhibitors for the treatment of these tumors (23); however, *de novo* or acquired treatment resistance has also been reported in several tumor models. Acquired CDK6 amplification has been found to promote breast cancer resistance to CDK4/6 inhibitors (24). In a pancreatic ductal adenocarcinoma cell model, CDK4/6 inhibition was associated with increased mTORC1 activity, which produces ATP accumulation at the mitochondrial level and induces drug resistance (25). In addition to kinase activity, CDK6 can participate in the transcriptional regulation of malignancy. For example, native CDK6 levels promote cell proliferation in T-cell lymphoma, yet forced overexpression of CDK6 protein inhibits cell proliferation by participating in positive transcriptional regulation of the tumor suppressor p16INK4a (26). In a chronic myeloid leukemia stem cell model, persistent application of CDK4/6 inhibitor resulted in CDK6-induced p53 mutation *via* a transcriptional regulation mechanism (27). These results suggest that CDK6 overexpression may be a double-edged sword for potential therapeutic targets in malignant tumors. Thus, ongoing research is currently focused either on the combination of CDK4/6 inhibitors and resistance-relevant antagonists (25, 28) or on precise medicine to avoid the occurrence of resistance (25, 29, 30). For example, research using the CRISPR-dCas9 screening approach in bladder cancer has indicated the beneficial effects of a combination of CDK4/6 inhibitors with some inhibitors against PI3K-Akt, Ras/MAPK, JAK/STAT, or Wnt signaling pathways in bladder cancer therapy (31).

*Ex vivo* tumor culture systems for drug sensitivity and resistance tests are an option for precision therapy for malignancy (32, 33). In this study, we identified that both high mRNA and protein levels of CDK6 predicted poor prognosis in BLCA cases through public database analysis and IHC staining of FFPE samples. We identified that the BLCA cell line with higher expression of CDK6 protein displayed greater sensitivity to CDK4/6 inhibitors. However, it is still arbitrary to judge whether patients with BLCA will be sensitive to CDK4/6 inhibitors using only mRNA evaluation or scoring the expression of CDK6 protein in FFPE specimens. Furthermore, passaging of BLCA cell lines cannot completely restore the tumor microenvironment. As fresh specimens of BLCA can be acquired after satisfying the need for diagnosis, we can attempt to develop an optimized and simplified procedure for tumor slices and various 3D culture systems to mimic the *in vivo* tumor microenvironment for precise prediction of the sensitivity and potential resistance to CDK4/6 inhibitors in the future.

In addition to BLCA, high transcriptional CDK6 mRNA also predicts poor prognosis in pancreatic adenocarcinoma, adrenocortical adenocarcinoma, uterine corpus endometrial carcinoma, lung adenocarcinoma, low-grade glioma, mesothelioma, and sarcoma from TCGA database analyzed using the UALCAN website (18). More interestingly, uterine corpus endometrial carcinoma displayed lower median transcriptional levels of CDK6 than normal tissues (*p* < 0.001) (data not shown), suggesting that malignancy displays high heterogeneity, and it is practical to evaluate IHC staining of CDK6 in these entities for precision therapy.

## Data Availability Statement

The raw RNA-sequencing data has been deposited in SRA database, BioProject number: PRJNA805290.

## Ethics Statement

The studies involving human participants were reviewed and approved by the Institutional Medical Ethics Review Board of the China-Japan Union Hospital of Jilin University in compliance with the Declaration of Helsinki. The reference number was 2021-KYLL-030003. The patients/participants provided their written informed consent to participate in this study. Written informed consent was obtained from the individual(s) for the publication of any potentially identifiable images or data included in this article. 

## Author Contributions

XJW developed the concept, analyzed the data and wrote the manuscript. XJW and RS designed the study. RS fulfilled cell proliferation experiments and western blot assay. XMW revised the manuscript. LCZ and YG were responsible for reviewing HE slides and scoring the IHC slides. SJY performed optimization of CDK6 antibody in IHC staining assay. LPW kindly provided technological supports and partial funding supports. All authors contributed to the article and approved the submitted version.

## Funding

This work is supported by the National Science and Technology Major Project of the Ministry of Science and Technology of China (subject grant No. 2017YFC0110105), the Young Scientists Fund of the National Natural Science Foundation of China (grant No. 81700198), the Science and Technology Development Project of Jilin Province (grant No. 20190701064GH), the Open Project of Key Laboratory of Tumor Immunology and Pathology (Army Medical University), Ministry of Education (2018jsz101), and Natural Science Foundation of Jilin province (20210101252JC).

## Conflict of Interest

The authors declare that the research was conducted in the absence of any commercial or financial relationships that could be construed as a potential conflict of interest.

## Publisher’s Note

All claims expressed in this article are solely those of the authors and do not necessarily represent those of their affiliated organizations, or those of the publisher, the editors and the reviewers. Any product that may be evaluated in this article, or claim that may be made by its manufacturer, is not guaranteed or endorsed by the publisher.
